# Aqua­(trifluoro­methane­sulfonato)­bis­(1,3,7-trimethyl­purine-2,6-dione)silver(I)

**DOI:** 10.1107/S1600536810035300

**Published:** 2010-09-04

**Authors:** Hadi D. Arman, Tyler Miller, Edward R. T. Tiekink

**Affiliations:** a Department of Chemistry, The University of Texas at San Antonio, One UTSA Circle, San Antonio, Texas 78249-0698, USA; b Department of Chemistry, University of Malaya, 50603 Kuala Lumpur, Malaysia

## Abstract

In the title compound, [Ag(CF_3_SO_3_)(C_8_H_10_N_4_O_2_)_2_(H_2_O)], the Ag^I^ atom is coordinated by two caffeine N atoms and, at longer distances, two O atoms of a coordinated water mol­ecule and the trifluoro­methane­sulfonate anion, resulting in an AgN_2_O_2_ seesaw geometry. The caffeine mol­ecules are roughly coplanar [dihedral angle = 5.81 (5)°]. In the crystal, mol­ecules self-assemble into a linear supra­molecular chain along the *c* axis *via* O—H⋯O hydrogen bonds involving the coordinated water moledcule and carbonyl O atoms. The packing is consolidated by weak C—H⋯O inter­actions.

## Related literature

For structural diversity in the supra­molecular structures of silver salts, see: Kundu *et al.* (2010 ▶). For a related Ag structure, see: Arman *et al.* (2010 ▶).
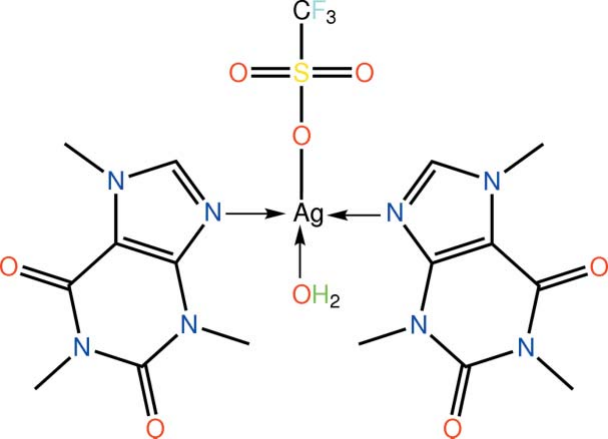


## Experimental

### 

#### Crystal data

[Ag(CF_3_SO_3_)(C_8_H_10_N_4_O_2_)_2_(H_2_O)]
*M* *_r_* = 663.36
Triclinic, 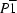

*a* = 8.9012 (10) Å
*b* = 10.0408 (8) Å
*c* = 15.457 (2) Å
α = 72.091 (7)°
β = 85.444 (9)°
γ = 63.672 (6)°
*V* = 1175.6 (2) Å^3^
*Z* = 2
Mo *K*α radiation
μ = 1.03 mm^−1^
*T* = 98 K
0.42 × 0.27 × 0.10 mm

#### Data collection

Rigaku AFC12/SATURN724 diffractometer
Absorption correction: multi-scan (*ABSCOR*; Higashi, 1995 ▶) *T*_min_ = 0.568, *T*_max_ = 1
7271 measured reflections
5323 independent reflections
5140 reflections with *I* > 2σ(*I*)
*R*_int_ = 0.020

#### Refinement

*R*[*F* ^2^ > 2σ(*F* ^2^)] = 0.034
*wR*(*F* ^2^) = 0.092
*S* = 1.14
5323 reflections
355 parameters
3 restraints
H-atom parameters constrained
Δρ_max_ = 0.63 e Å^−3^
Δρ_min_ = −0.86 e Å^−3^

### 

Data collection: *CrystalClear* (Molecular Structure Corporation & Rigaku, 2005 ▶); cell refinement: *CrystalClear*; data reduction: *CrystalClear*; program(s) used to solve structure: *SHELXS97* (Sheldrick, 2008 ▶); program(s) used to refine structure: *SHELXL97* (Sheldrick, 2008 ▶); molecular graphics: *ORTEP-3* (Farrugia, 1997 ▶) and *DIAMOND* (Brandenburg, 2006 ▶); software used to prepare material for publication: *publCIF* (Westrip, 2010 ▶).

## Supplementary Material

Crystal structure: contains datablocks global, I. DOI: 10.1107/S1600536810035300/hb5627sup1.cif

Structure factors: contains datablocks I. DOI: 10.1107/S1600536810035300/hb5627Isup2.hkl

Additional supplementary materials:  crystallographic information; 3D view; checkCIF report

## Figures and Tables

**Table:** 

Ag—N7	2.213 (2)
Ag—N3	2.218 (2)
Ag—O1w	2.4347 (19)
Ag—O7	2.5591 (19)

**Table:** 

N7—Ag—N3	165.48 (8)
N7—Ag—O1w	98.70 (8)
N3—Ag—O1w	95.81 (7)
N7—Ag—O7	90.01 (7)
N3—Ag—O7	88.93 (7)
O1w—Ag—O7	92.39 (7)

**Table 2:** Hydrogen-bond geometry (Å, °)

*D*—H⋯*A*	*D*—H	H⋯*A*	*D*⋯*A*	*D*—H⋯*A*
O1w—H1w⋯O1^i^	0.84	1.89	2.724 (3)	170
O1w—H2w⋯O3^ii^	0.84	1.89	2.701 (3)	163
C10—H10c⋯O1w^ii^	0.98	2.55	3.428 (4)	149
C15—H15c⋯O4^ii^	0.98	2.60	3.382 (4)	137
C4—H4b⋯O6^iii^	0.98	2.41	3.252 (4)	144
C12—H12c⋯O5^iii^	0.98	2.36	3.278 (4)	155

Symmetry codes: (i) 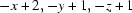; (ii) 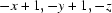; (iii) 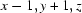.

**Fig. 1.**
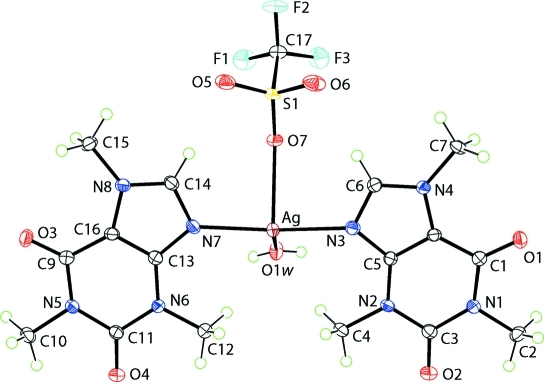
Asymmetric unit in the structure of (I) showing displacement ellipsoids at the 50% probability level.

**Fig. 2.**
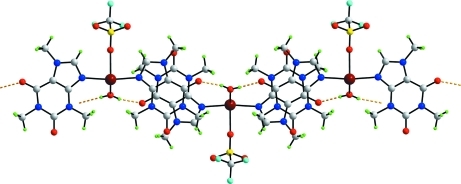
Portion of the supramolecular chain aligned along the c axis in (I). The O—H···O hydrogen bonds are shown as orange dashed lines.

**Fig. 3.**
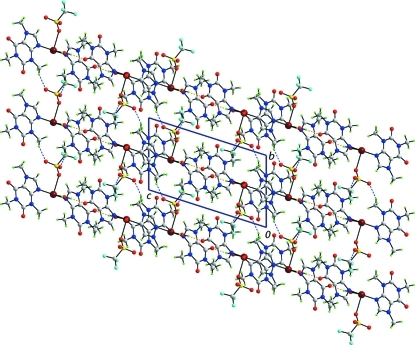
A view in projection down the a axis of the crystal packing in (I). The O—H···O hydrogen bonds and C—H···O contacts are shown as orange and blue dashed lines, respectively.

## supplementary crystallographic information

### Comment

As a continuation of recent structural studies on silver salts (Arman *et al.*, 2010), of fascination owing to the structural diversity of their supramolecular structures (Kundu *et al.*, 2010), the title compound, (I), was isolated and characterized.

The Ag atom in (I) is coordinated by a water molecule, two N atoms derived from two caffeine molecules and an O atom from the trifluoromethanesulfonate anion, Fig. 1. While the Ag—N bond distances are experimentally equivalent, they are shorter than the Ag—O(water) and even longer Ag—O(trifluoromethanesulfonate) distances, Table 1. Reflecting the disparity in the Ag—*X* bond distances, the N_2_O_2_ coordination geometry is highly distorted tetrahedral owing to the dominanace of the Ag—N bonds that are almost diagonally opposite [N3—Ag—N7 = 165.48 (8) °]. Each of the N3- and N7-caffeine rings is planar [r.m.s. deviation of the 14 non-hydrogen atoms = 0.013 and 0.029 Å, respectively] and are almost co-planar as seen in the dihedral angle formed between them of 5.81 (5) °.

In the crystal packing, centrosymmetrically related molecules associate *via* O—H···O hydrogen bonds formed between the coordinated water molecule and carbonyl-O, Fig. 2 and Table 2. This arrangement is stabilized by C—H···O interactions involving the O1w and O4 atoms as acceptors, Table 1, and π···π [ring centroid(N1,N2,C1,C3,C5,C8)···centroid(N1,N2,C1,C3,C5,C8)^i^ = 3.5605 (16) ° for *i*: 2 - *x*, 1 - *y*, 1 - *z*] contacts. The primary interactions linking the resulting supramolecular chains aligned along the *c* axis are of the type C—H···O, Fig. 3 and Table 1.

### Experimental

Caffeine (Analytical & Research Chemical Company, 0.015 g, 0.08 mmol) was dissolved in 5 ml of ethanol and silver trifluoromethanesulfonate (ACROS, 0.012 g, 0.04 mmol) also dissolved in 5 ml of ethanol was added to this. The resulting solution was gently heated and allowed to stand for slow evaporation, which afforded colourless blocks of (I) after 10 days; m. pt: 447–451 K. IR (cm^-1^): ν(O—H) 3454, ν(C═O) 1700, ν(C═N)1549, ν(C—F) 1156, ν(S—O) 1028.

### Refinement

C-bound H-atoms were placed in calculated positions (C—H 0.95–0.98 Å) and were included in the refinement in the riding model approximation with *U*_iso_(H) set to 1.2–1.5*U*_eq_(C). The O—H atoms were refined with O—H = 0.8400±0.0001, and with *U*_iso_(H) set to 1.5*U*_eq_(C)

### Crystal data

**Table:**

[Ag(CF_3_SO_3_)(C_8_H_10_N_4_O_2_)_2_(H_2_O)]	*Z* = 2
*M**_r_* = 663.36	*F*(000) = 668
Triclinic, *P*1	*D*_x_ = 1.874 Mg m^−^^3^
Hall symbol: -P 1	Mo *K*α radiation, λ = 0.71069 Å
*a* = 8.9012 (10) Å	Cell parameters from 3724 reflections
*b* = 10.0408 (8) Å	θ = 2.4–40.6°
*c* = 15.457 (2) Å	µ = 1.03 mm^−^^1^
α = 72.091 (7)°	*T* = 98 K
β = 85.444 (9)°	Block, colorless
γ = 63.672 (6)°	0.42 × 0.27 × 0.10 mm
*V* = 1175.6 (2) Å^3^	

### Data collection

**Table:**

Rigaku AFC12K/SATURN724 diffractometer	5323 independent reflections
Radiation source: fine-focus sealed tube	5140 reflections with *I* > 2σ(*I*)
graphite	*R*_int_ = 0.020
ω scans	θ_max_ = 27.5°, θ_min_ = 2.4°
Absorption correction: multi-scan (*ABSCOR*; Higashi, 1995)	*h* = −11→11
*T*_min_ = 0.568, *T*_max_ = 1	*k* = −13→11
7271 measured reflections	*l* = −20→19

### Refinement

**Table:**

Refinement on *F*^2^	Primary atom site location: structure-invariant direct methods
Least-squares matrix: full	Secondary atom site location: difference Fourier map
*R*[*F*^2^ > 2σ(*F*^2^)] = 0.034	Hydrogen site location: inferred from neighbouring sites
*wR*(*F*^2^) = 0.092	H-atom parameters constrained
*S* = 1.14	*w* = 1/[σ^2^(*F*_o_^2^) + (0.0466*P*)^2^ + 1.1737*P*] where *P* = (*F*_o_^2^ + 2*F*_c_^2^)/3
5323 reflections	(Δ/σ)_max_ = 0.001
355 parameters	Δρ_max_ = 0.63 e Å^−^^3^
3 restraints	Δρ_min_ = −0.86 e Å^−^^3^

### Special details

**Table:**

Geometry. All e.s.d.'s (except the e.s.d. in the dihedral angle between two l.s. planes) are estimated using the full covariance matrix. The cell e.s.d.'s are taken into account individually in the estimation of e.s.d.'s in distances, angles and torsion angles; correlations between e.s.d.'s in cell parameters are only used when they are defined by crystal symmetry. An approximate (isotropic) treatment of cell e.s.d.'s is used for estimating e.s.d.'s involving l.s. planes.
Refinement. Refinement of *F*^2^ against ALL reflections. The weighted *R*-factor *wR* and goodness of fit *S* are based on *F*^2^, conventional *R*-factors *R* are based on *F*, with *F* set to zero for negative *F*^2^. The threshold expression of *F*^2^ > σ(*F*^2^) is used only for calculating *R*-factors(gt) *etc*. and is not relevant to the choice of reflections for refinement. *R*-factors based on *F*^2^ are statistically about twice as large as those based on *F*, and *R*- factors based on ALL data will be even larger.

### Fractional atomic coordinates and isotropic or equivalent isotropic displacement parameters (Å^2^)

**Table:**

	*x*	*y*	*z*	*U*_iso_*/*U*_eq_	
Ag	0.92178 (2)	0.48844 (2)	0.188475 (12)	0.01653 (8)	
S1	1.24387 (8)	0.07772 (7)	0.21533 (4)	0.01613 (13)	
F1	1.1161 (2)	−0.1212 (2)	0.27088 (15)	0.0355 (4)	
F2	1.3860 (2)	−0.2279 (2)	0.27639 (14)	0.0331 (4)	
F3	1.2548 (3)	−0.1155 (2)	0.37600 (12)	0.0356 (4)	
O1	1.2942 (2)	0.5290 (2)	0.54121 (13)	0.0215 (4)	
O2	0.7737 (3)	0.9110 (2)	0.41597 (14)	0.0233 (4)	
O3	0.5572 (2)	0.5224 (2)	−0.19291 (13)	0.0217 (4)	
O4	0.3195 (2)	0.9238 (2)	−0.06788 (13)	0.0200 (4)	
O1w	0.6921 (2)	0.4768 (3)	0.28220 (13)	0.0225 (4)	
H1w	0.6922	0.4669	0.3382	0.034*	
H2w	0.6035	0.4812	0.2635	0.034*	
O5	1.2439 (3)	0.0652 (3)	0.12484 (14)	0.0292 (5)	
O6	1.3918 (3)	0.0794 (2)	0.24474 (17)	0.0295 (5)	
O7	1.0872 (2)	0.1910 (2)	0.23664 (13)	0.0196 (4)	
N1	1.0320 (3)	0.7175 (3)	0.47948 (15)	0.0161 (4)	
N2	0.9007 (3)	0.7135 (3)	0.35260 (15)	0.0160 (4)	
N3	1.0696 (3)	0.4918 (3)	0.29602 (15)	0.0170 (4)	
N4	1.2894 (3)	0.3795 (3)	0.39758 (15)	0.0160 (4)	
N5	0.4349 (3)	0.7199 (3)	−0.12752 (15)	0.0160 (4)	
N6	0.5649 (3)	0.7186 (3)	0.00204 (14)	0.0155 (4)	
N7	0.8288 (3)	0.4857 (3)	0.06071 (15)	0.0172 (4)	
N8	0.8324 (3)	0.3657 (3)	−0.04021 (15)	0.0166 (4)	
C1	1.1779 (3)	0.5806 (3)	0.48418 (17)	0.0156 (5)	
C2	1.0253 (4)	0.7989 (3)	0.54543 (18)	0.0208 (5)	
H2A	1.1136	0.8343	0.5346	0.031*	
H2B	1.0427	0.7275	0.6075	0.031*	
H2C	0.9153	0.8892	0.5381	0.031*	
C3	0.8935 (3)	0.7894 (3)	0.41529 (18)	0.0169 (5)	
C4	0.7618 (3)	0.7835 (3)	0.28352 (19)	0.0206 (5)	
H4A	0.7994	0.8207	0.2235	0.031*	
H4B	0.6682	0.8714	0.2978	0.031*	
H4C	0.7247	0.7052	0.2829	0.031*	
C5	1.0406 (3)	0.5789 (3)	0.35362 (17)	0.0143 (5)	
C6	1.2221 (3)	0.3717 (3)	0.32635 (18)	0.0168 (5)	
H6	1.2761	0.2898	0.2997	0.020*	
C7	1.4535 (3)	0.2678 (3)	0.44590 (19)	0.0207 (5)	
H7A	1.5136	0.1906	0.4138	0.031*	
H7B	1.4372	0.2147	0.5081	0.031*	
H7C	1.5193	0.3234	0.4481	0.031*	
C8	1.1738 (3)	0.5129 (3)	0.41647 (17)	0.0147 (5)	
C9	0.5606 (3)	0.5776 (3)	−0.13270 (17)	0.0154 (5)	
C10	0.2981 (3)	0.8070 (3)	−0.19966 (19)	0.0207 (5)	
H10A	0.3385	0.8553	−0.2556	0.031*	
H10B	0.2040	0.8883	−0.1803	0.031*	
H10C	0.2605	0.7354	−0.2111	0.031*	
C11	0.4333 (3)	0.7971 (3)	−0.06454 (17)	0.0154 (5)	
C12	0.5725 (3)	0.7927 (3)	0.06883 (18)	0.0202 (5)	
H12A	0.6628	0.8253	0.0554	0.030*	
H12B	0.5948	0.7182	0.1302	0.030*	
H12C	0.4651	0.8842	0.0655	0.030*	
C13	0.6907 (3)	0.5769 (3)	0.00148 (17)	0.0149 (5)	
C14	0.9101 (3)	0.3590 (3)	0.03247 (17)	0.0174 (5)	
H14	1.0126	0.2734	0.0613	0.021*	
C15	0.8874 (4)	0.2440 (3)	−0.08522 (19)	0.0203 (5)	
H15A	1.0015	0.1650	−0.0615	0.030*	
H15B	0.8865	0.2908	−0.1511	0.030*	
H15C	0.8110	0.1950	−0.0731	0.030*	
C16	0.6894 (3)	0.5071 (3)	−0.06174 (17)	0.0159 (5)	
C17	1.2518 (3)	−0.1069 (3)	0.28803 (19)	0.0200 (5)	

### Atomic displacement parameters (Å^2^)

**Table:**

	*U*^11^	*U*^22^	*U*^33^	*U*^12^	*U*^13^	*U*^23^
Ag	0.01694 (11)	0.01610 (12)	0.01605 (11)	−0.00609 (8)	−0.00257 (7)	−0.00519 (8)
S1	0.0131 (3)	0.0120 (3)	0.0189 (3)	−0.0024 (2)	0.0012 (2)	−0.0038 (2)
F1	0.0277 (9)	0.0307 (10)	0.0546 (12)	−0.0190 (8)	0.0034 (9)	−0.0122 (9)
F2	0.0281 (9)	0.0136 (8)	0.0456 (11)	−0.0009 (7)	0.0068 (8)	−0.0071 (8)
F3	0.0466 (12)	0.0338 (10)	0.0181 (8)	−0.0151 (9)	0.0034 (8)	−0.0013 (7)
O1	0.0206 (10)	0.0245 (10)	0.0188 (9)	−0.0079 (8)	−0.0022 (8)	−0.0080 (8)
O2	0.0214 (10)	0.0174 (10)	0.0259 (10)	−0.0028 (8)	−0.0015 (8)	−0.0078 (8)
O3	0.0203 (9)	0.0267 (11)	0.0209 (10)	−0.0088 (8)	−0.0008 (8)	−0.0129 (8)
O4	0.0192 (9)	0.0153 (9)	0.0216 (9)	−0.0038 (7)	−0.0007 (7)	−0.0060 (7)
O1w	0.0198 (10)	0.0333 (11)	0.0189 (9)	−0.0131 (9)	0.0027 (8)	−0.0122 (9)
O5	0.0284 (11)	0.0232 (11)	0.0189 (10)	0.0030 (9)	0.0027 (8)	−0.0059 (8)
O6	0.0157 (9)	0.0203 (10)	0.0511 (14)	−0.0071 (8)	−0.0003 (9)	−0.0095 (10)
O7	0.0154 (9)	0.0161 (9)	0.0239 (10)	−0.0021 (7)	0.0002 (7)	−0.0089 (8)
N1	0.0174 (10)	0.0149 (10)	0.0145 (10)	−0.0062 (9)	−0.0006 (8)	−0.0037 (8)
N2	0.0144 (10)	0.0118 (10)	0.0184 (10)	−0.0037 (8)	−0.0032 (8)	−0.0024 (8)
N3	0.0152 (10)	0.0150 (10)	0.0205 (11)	−0.0066 (9)	−0.0003 (8)	−0.0051 (9)
N4	0.0135 (10)	0.0146 (10)	0.0192 (10)	−0.0049 (8)	−0.0003 (8)	−0.0061 (8)
N5	0.0164 (10)	0.0142 (10)	0.0153 (10)	−0.0059 (8)	−0.0016 (8)	−0.0027 (8)
N6	0.0179 (10)	0.0136 (10)	0.0138 (10)	−0.0053 (8)	0.0002 (8)	−0.0048 (8)
N7	0.0161 (10)	0.0168 (11)	0.0139 (10)	−0.0037 (9)	−0.0013 (8)	−0.0032 (8)
N8	0.0169 (10)	0.0129 (10)	0.0165 (10)	−0.0041 (8)	0.0009 (8)	−0.0037 (8)
C1	0.0161 (12)	0.0153 (12)	0.0160 (11)	−0.0084 (10)	0.0016 (9)	−0.0035 (9)
C2	0.0258 (14)	0.0180 (13)	0.0182 (12)	−0.0079 (11)	0.0015 (10)	−0.0078 (10)
C3	0.0169 (12)	0.0150 (12)	0.0184 (12)	−0.0082 (10)	0.0005 (10)	−0.0025 (10)
C4	0.0163 (12)	0.0160 (12)	0.0250 (13)	−0.0027 (10)	−0.0067 (10)	−0.0049 (10)
C5	0.0135 (11)	0.0141 (11)	0.0163 (11)	−0.0071 (9)	0.0006 (9)	−0.0042 (9)
C6	0.0161 (12)	0.0159 (12)	0.0188 (12)	−0.0065 (10)	0.0004 (9)	−0.0062 (10)
C7	0.0118 (11)	0.0196 (13)	0.0239 (13)	−0.0009 (10)	−0.0030 (10)	−0.0059 (11)
C8	0.0143 (11)	0.0123 (11)	0.0169 (11)	−0.0052 (9)	0.0008 (9)	−0.0046 (9)
C9	0.0141 (11)	0.0153 (12)	0.0164 (11)	−0.0068 (9)	0.0027 (9)	−0.0044 (9)
C10	0.0181 (12)	0.0188 (13)	0.0227 (13)	−0.0065 (10)	−0.0062 (10)	−0.0037 (11)
C11	0.0164 (11)	0.0142 (12)	0.0141 (11)	−0.0068 (9)	0.0007 (9)	−0.0022 (9)
C12	0.0216 (13)	0.0179 (13)	0.0186 (12)	−0.0053 (10)	−0.0015 (10)	−0.0070 (10)
C13	0.0155 (11)	0.0148 (12)	0.0138 (11)	−0.0074 (10)	0.0018 (9)	−0.0025 (9)
C14	0.0157 (12)	0.0163 (12)	0.0162 (12)	−0.0044 (10)	−0.0013 (9)	−0.0033 (10)
C15	0.0230 (13)	0.0165 (12)	0.0213 (13)	−0.0067 (10)	0.0034 (10)	−0.0093 (10)
C16	0.0155 (12)	0.0128 (11)	0.0158 (11)	−0.0036 (9)	0.0014 (9)	−0.0039 (9)
C17	0.0183 (12)	0.0155 (12)	0.0232 (13)	−0.0054 (10)	0.0031 (10)	−0.0054 (10)

### Geometric parameters (Å, °)

**Table:**

Ag—N7	2.213 (2)	N6—C11	1.388 (3)
Ag—N3	2.218 (2)	N6—C12	1.467 (3)
Ag—O1w	2.4347 (19)	N7—C14	1.344 (3)
Ag—O7	2.5591 (19)	N7—C13	1.361 (3)
S1—O6	1.436 (2)	N8—C14	1.335 (3)
S1—O5	1.442 (2)	N8—C16	1.386 (3)
S1—O7	1.4482 (19)	N8—C15	1.468 (3)
S1—C17	1.822 (3)	C1—C8	1.423 (4)
F1—C17	1.334 (3)	C2—H2A	0.9800
F2—C17	1.322 (3)	C2—H2B	0.9800
F3—C17	1.338 (3)	C2—H2C	0.9800
O1—C1	1.227 (3)	C4—H4A	0.9800
O2—C3	1.216 (3)	C4—H4B	0.9800
O3—C9	1.229 (3)	C4—H4C	0.9800
O4—C11	1.213 (3)	C5—C8	1.371 (4)
O1w—H1w	0.8401	C6—H6	0.9500
O1w—H2w	0.8400	C7—H7A	0.9800
N1—C1	1.397 (3)	C7—H7B	0.9800
N1—C3	1.411 (3)	C7—H7C	0.9800
N1—C2	1.473 (3)	C9—C16	1.422 (4)
N2—C5	1.369 (3)	C10—H10A	0.9800
N2—C3	1.388 (3)	C10—H10B	0.9800
N2—C4	1.463 (3)	C10—H10C	0.9800
N3—C6	1.349 (3)	C12—H12A	0.9800
N3—C5	1.365 (3)	C12—H12B	0.9800
N4—C6	1.333 (3)	C12—H12C	0.9800
N4—C8	1.381 (3)	C13—C16	1.370 (4)
N4—C7	1.468 (3)	C14—H14	0.9500
N5—C9	1.392 (3)	C15—H15A	0.9800
N5—C11	1.415 (3)	C15—H15B	0.9800
N5—C10	1.471 (3)	C15—H15C	0.9800
N6—C13	1.368 (3)		
			
N7—Ag—N3	165.48 (8)	H4A—C4—H4C	109.5
N7—Ag—O1w	98.70 (8)	H4B—C4—H4C	109.5
N3—Ag—O1w	95.81 (7)	N3—C5—N2	127.2 (2)
N7—Ag—O7	90.01 (7)	N3—C5—C8	110.6 (2)
N3—Ag—O7	88.93 (7)	N2—C5—C8	122.2 (2)
O1w—Ag—O7	92.39 (7)	N4—C6—N3	113.1 (2)
O6—S1—O5	115.21 (14)	N4—C6—H6	123.5
O6—S1—O7	114.71 (13)	N3—C6—H6	123.5
O5—S1—O7	114.71 (12)	N4—C7—H7A	109.5
O6—S1—C17	104.22 (13)	N4—C7—H7B	109.5
O5—S1—C17	103.14 (13)	H7A—C7—H7B	109.5
O7—S1—C17	102.51 (12)	N4—C7—H7C	109.5
Ag—O1w—H1w	123.9	H7A—C7—H7C	109.5
Ag—O1w—H2w	125.1	H7B—C7—H7C	109.5
H1W—O1w—H2w	111.1	C5—C8—N4	106.2 (2)
S1—O7—Ag	136.78 (12)	C5—C8—C1	122.5 (2)
C1—N1—C3	126.5 (2)	N4—C8—C1	131.3 (2)
C1—N1—C2	116.8 (2)	O3—C9—N5	122.4 (2)
C3—N1—C2	116.7 (2)	O3—C9—C16	125.2 (2)
C5—N2—C3	119.6 (2)	N5—C9—C16	112.4 (2)
C5—N2—C4	121.2 (2)	N5—C10—H10A	109.5
C3—N2—C4	119.1 (2)	N5—C10—H10B	109.5
C6—N3—C5	103.9 (2)	H10A—C10—H10B	109.5
C6—N3—Ag	119.15 (18)	N5—C10—H10C	109.5
C5—N3—Ag	136.34 (18)	H10A—C10—H10C	109.5
C6—N4—C8	106.3 (2)	H10B—C10—H10C	109.5
C6—N4—C7	126.5 (2)	O4—C11—N6	122.3 (2)
C8—N4—C7	127.3 (2)	O4—C11—N5	121.0 (2)
C9—N5—C11	126.4 (2)	N6—C11—N5	116.6 (2)
C9—N5—C10	117.3 (2)	N6—C12—H12A	109.5
C11—N5—C10	116.0 (2)	N6—C12—H12B	109.5
C13—N6—C11	119.6 (2)	H12A—C12—H12B	109.5
C13—N6—C12	120.8 (2)	N6—C12—H12C	109.5
C11—N6—C12	119.6 (2)	H12A—C12—H12C	109.5
C14—N7—C13	104.0 (2)	H12B—C12—H12C	109.5
C14—N7—Ag	119.11 (17)	N7—C13—N6	127.1 (2)
C13—N7—Ag	136.45 (18)	N7—C13—C16	110.8 (2)
C14—N8—C16	106.0 (2)	N6—C13—C16	122.1 (2)
C14—N8—C15	126.0 (2)	N8—C14—N7	113.2 (2)
C16—N8—C15	127.9 (2)	N8—C14—H14	123.4
O1—C1—N1	121.8 (2)	N7—C14—H14	123.4
O1—C1—C8	125.7 (2)	N8—C15—H15A	109.5
N1—C1—C8	112.5 (2)	N8—C15—H15B	109.5
N1—C2—H2A	109.5	H15A—C15—H15B	109.5
N1—C2—H2B	109.5	N8—C15—H15C	109.5
H2A—C2—H2B	109.5	H15A—C15—H15C	109.5
N1—C2—H2C	109.5	H15B—C15—H15C	109.5
H2A—C2—H2C	109.5	C13—C16—N8	106.0 (2)
H2B—C2—H2C	109.5	C13—C16—C9	122.8 (2)
O2—C3—N2	122.2 (2)	N8—C16—C9	131.2 (2)
O2—C3—N1	121.2 (2)	F2—C17—F1	108.1 (2)
N2—C3—N1	116.6 (2)	F2—C17—F3	107.9 (2)
N2—C4—H4A	109.5	F1—C17—F3	107.0 (2)
N2—C4—H4B	109.5	F2—C17—S1	112.01 (19)
H4A—C4—H4B	109.5	F1—C17—S1	110.82 (19)
N2—C4—H4C	109.5	F3—C17—S1	110.9 (2)
			
O6—S1—O7—Ag	66.6 (2)	O1—C1—C8—C5	−178.7 (2)
O5—S1—O7—Ag	−70.1 (2)	N1—C1—C8—C5	0.9 (3)
C17—S1—O7—Ag	178.87 (16)	O1—C1—C8—N4	0.7 (5)
N7—Ag—O7—S1	78.84 (17)	N1—C1—C8—N4	−179.7 (2)
N3—Ag—O7—S1	−86.68 (17)	C11—N5—C9—O3	−175.7 (2)
O1w—Ag—O7—S1	177.55 (17)	C10—N5—C9—O3	−2.2 (4)
N7—Ag—N3—C6	−62.4 (4)	C11—N5—C9—C16	4.1 (4)
O1w—Ag—N3—C6	115.84 (19)	C10—N5—C9—C16	177.6 (2)
O7—Ag—N3—C6	23.54 (19)	C13—N6—C11—O4	−179.2 (2)
N7—Ag—N3—C5	128.5 (3)	C12—N6—C11—O4	−2.8 (4)
O1w—Ag—N3—C5	−53.3 (3)	C13—N6—C11—N5	2.8 (3)
O7—Ag—N3—C5	−145.6 (2)	C12—N6—C11—N5	179.2 (2)
N3—Ag—N7—C14	67.4 (4)	C9—N5—C11—O4	177.6 (2)
O1w—Ag—N7—C14	−110.8 (2)	C10—N5—C11—O4	4.1 (4)
O7—Ag—N7—C14	−18.4 (2)	C9—N5—C11—N6	−4.3 (4)
N3—Ag—N7—C13	−121.7 (3)	C10—N5—C11—N6	−177.8 (2)
O1w—Ag—N7—C13	60.1 (3)	C14—N7—C13—N6	178.6 (2)
O7—Ag—N7—C13	152.5 (3)	Ag—N7—C13—N6	6.8 (4)
C3—N1—C1—O1	178.0 (2)	C14—N7—C13—C16	−0.5 (3)
C2—N1—C1—O1	1.0 (4)	Ag—N7—C13—C16	−172.28 (19)
C3—N1—C1—C8	−1.7 (3)	C11—N6—C13—N7	179.3 (2)
C2—N1—C1—C8	−178.6 (2)	C12—N6—C13—N7	2.9 (4)
C5—N2—C3—O2	178.4 (2)	C11—N6—C13—C16	−1.7 (4)
C4—N2—C3—O2	1.6 (4)	C12—N6—C13—C16	−178.1 (2)
C5—N2—C3—N1	−2.3 (3)	C16—N8—C14—N7	0.1 (3)
C4—N2—C3—N1	−179.2 (2)	C15—N8—C14—N7	−178.3 (2)
C1—N1—C3—O2	−178.3 (2)	C13—N7—C14—N8	0.2 (3)
C2—N1—C3—O2	−1.4 (4)	Ag—N7—C14—N8	173.80 (17)
C1—N1—C3—N2	2.4 (4)	N7—C13—C16—N8	0.5 (3)
C2—N1—C3—N2	179.3 (2)	N6—C13—C16—N8	−178.7 (2)
C6—N3—C5—N2	−179.1 (2)	N7—C13—C16—C9	−179.1 (2)
Ag—N3—C5—N2	−8.8 (4)	N6—C13—C16—C9	1.7 (4)
C6—N3—C5—C8	0.6 (3)	C14—N8—C16—C13	−0.3 (3)
Ag—N3—C5—C8	170.88 (18)	C15—N8—C16—C13	178.0 (2)
C3—N2—C5—N3	−178.5 (2)	C14—N8—C16—C9	179.2 (3)
C4—N2—C5—N3	−1.7 (4)	C15—N8—C16—C9	−2.5 (4)
C3—N2—C5—C8	1.8 (4)	O3—C9—C16—C13	177.1 (2)
C4—N2—C5—C8	178.6 (2)	N5—C9—C16—C13	−2.7 (4)
C8—N4—C6—N3	0.5 (3)	O3—C9—C16—N8	−2.4 (5)
C7—N4—C6—N3	−179.7 (2)	N5—C9—C16—N8	177.8 (3)
C5—N3—C6—N4	−0.7 (3)	O6—S1—C17—F2	−62.1 (2)
Ag—N3—C6—N4	−172.98 (17)	O5—S1—C17—F2	58.6 (2)
N3—C5—C8—N4	−0.4 (3)	O7—S1—C17—F2	178.0 (2)
N2—C5—C8—N4	179.4 (2)	O6—S1—C17—F1	177.1 (2)
N3—C5—C8—C1	179.2 (2)	O5—S1—C17—F1	−62.2 (2)
N2—C5—C8—C1	−1.1 (4)	O7—S1—C17—F1	57.3 (2)
C6—N4—C8—C5	0.0 (3)	O6—S1—C17—F3	58.5 (2)
C7—N4—C8—C5	−179.9 (2)	O5—S1—C17—F3	179.17 (19)
C6—N4—C8—C1	−179.5 (3)	O7—S1—C17—F3	−61.4 (2)
C7—N4—C8—C1	0.6 (4)		

### Hydrogen-bond geometry (Å, °)

**Table:**

*D*—H···*A*	*D*—H	H···*A*	*D*···*A*	*D*—H···*A*
O1w—H1w···O1^i^	0.84	1.89	2.724 (3)	170
O1w—H2w···O3^ii^	0.84	1.89	2.701 (3)	163
C10—H10c···O1w^ii^	0.98	2.55	3.428 (4)	149
C15—H15c···O4^ii^	0.98	2.60	3.382 (4)	137
C4—H4b···O6^iii^	0.98	2.41	3.252 (4)	144
C12—H12c···O5^iii^	0.98	2.36	3.278 (4)	155

Symmetry codes: (i) −*x*+2, −*y*+1, −*z*+1; (ii) −*x*+1, −*y*+1, −*z*; (iii) *x*−1, *y*+1, *z*.
